# Redox regulation of chloroplast metabolism

**DOI:** 10.1093/plphys/kiaa062

**Published:** 2020-12-18

**Authors:** Francisco Javier Cejudo, María-Cruz González, Juan Manuel Pérez-Ruiz

**Affiliations:** Instituto de Bioquímica Vegetal y Fotosíntesis, Universidad de Sevilla—Consejo Superior de Investigaciones Científicas, Avda. Américo Vespucio 49, 41092 Sevilla, Spain

## Abstract

Regulation of enzyme activity based on thiol-disulfide exchange is a regulatory mechanism in which the protein disulfide reductase activity of thioredoxins (TRXs) plays a central role. Plant chloroplasts are equipped with a complex set of up to 20 TRXs and TRX-like proteins, the activity of which is supported by reducing power provided by photosynthetically reduced ferredoxin (FDX) with the participation of a FDX-dependent TRX reductase (FTR). Therefore, the FDX–FTR–TRXs pathway allows the regulation of redox-sensitive chloroplast enzymes in response to light. In addition, chloroplasts contain an NADPH-dependent redox system, termed NTRC, which allows the use of NADPH in the redox network of these organelles. Genetic approaches using mutants of Arabidopsis (*Arabidopsis thaliana*) in combination with biochemical and physiological studies have shown that both redox systems, NTRC and FDX-FTR-TRXs, participate in fine-tuning chloroplast performance in response to changes in light intensity. Moreover, these studies revealed the participation of 2-Cys peroxiredoxin (2-Cys PRX), a thiol-dependent peroxidase, in the control of the reducing activity of chloroplast TRXs as well as in the rapid oxidation of stromal enzymes upon darkness. In this review, we provide an update on recent findings regarding the redox regulatory network of plant chloroplasts, focusing on the functional relationship of 2-Cys PRXs with NTRC and the FDX–FTR–TRXs redox systems for fine-tuning chloroplast performance in response to changes in light intensity and darkness. Finally, we consider redox regulation as an additional layer of control of the signaling function of the chloroplast.

## Teaser

Thiol-dependent redox regulatory and antioxidant systems act concertedly to modulate chloroplast metabolism and signaling function.

**Advances**

- Plant chloroplasts harbor a complex redox network composed of the FDX–FTR–TRXs pathway, linking redox regulation to light, and NTRC, an NADPH-dependent system required for the activity of TRXs. Both systems adjust chloroplast performance to environmental cues.
- A relevant function of NTRC is redox control of 2-Cys PRXs, which maintains the reductive activity of chloroplast TRXs in the light. The NTRC–2-Cys PRXs redox system helps fine-tune the redox state of chloroplast enzymes thereby adjusting photosynthetic performance to changes in light.
- 2-Cys PRXs participate in the rapid oxidative inactivation of chloroplast enzymes in the dark, mediating the transfer of reducing equivalents from reduced enzymes, via TRXs, to hydrogen peroxide.
- Involvement of redox regulation in chloroplast retrograde signaling modulates early stages of plant development and response to environmental stress.

## Introduction

As a process that involves the transport of electrons in the presence of oxygen, photosynthesis inevitably produces reactive oxygen species (ROS), which may cause oxidative damage; therefore, photosynthetic performance is tightly linked to antioxidant systems that maintain ROS under non-toxic concentrations. Different environmental conditions lead to an imbalance of ROS production and scavenging causing oxidative stress, which has a negative effect on plant growth. Beside their potential toxic effect, ROS has a very important signaling function acting as systemic signals that affect the pattern of expression of a large number of genes, hence coordinating plant response to environmental stress conditions (Zandalinas et al., 2020). ROS signaling depends on the capacity to provoke post-translational modifications in proteins, which is emerging as an important mechanism for the adaptability of plants to the environment (Waszczak et al., 2015; Young et al., 2019). In this regard, cysteine, due to the high reactivity of the thiolate group (Ferrer-Sueta et al., 2011), is the target of many of these modifications. Of interest for this review, cysteine thiolate reacts with hydrogen peroxide being oxidized to sulfenic acid (-SO^−^) which, under oxidizing conditions, can be further oxidized to sulfinic (-${\text{SO}}_{2}^{-}$) or even sulfonic acid (-${\text{SO}}_{3}^{-}$). In addition, cysteines can form intra- or intermolecular disulfides that have deep effects on protein conformation and activity.

Thiol–disulfide interchange constitutes the basis of redox regulation, which is a universal regulatory mechanism since redox-sensitive proteins are found in all organisms (Balsera and Buchanan, 2019). While the mechanism of oxidative disulfide formation in vivo has remained poorly understood, the participation of thioredoxins (TRXs) in disulfide reduction is well established. TRXs are small polypeptides of 12–14 kDa with a characteristic folding, the so-called TRX-fold, and an active site, formed by the signature WCG/PPC, localized at the protein surface (Holmgren, 1995). The reductive activation of target enzymes catalyzed by the disulfide reductase activity of TRXs requires reducing power, which in heterotrophic organisms is provided by NADPH with the participation of an NADPH-dependent TRX reductase (NTR; Jacquot et al., 2009). In plant chloroplasts, redox regulation shows relevant differences when compared with non-photosynthetic organisms (Cejudo et al., 2019). The complex set of up to 20 TRX isoforms of chloroplasts (Geigenberger et al., 2017; Nikkanen and Rintamäki, 2019; Zaffagnini et al., 2019) relies on ferredoxin (FDX) reduced by the photosynthetic electron transport chain through the activity of a FDX-dependent TRX reductase (FTR; Schürmann and Buchanan, 2008). Approaches for trapping TRX interacting proteins combined with mass spectrometry have identified more than 400 putative TRX targets including many chloroplast enzymes (Montrichard et al., 2009), which means that virtually any process occurring in the organelle is under redox regulation.

Beside the FDX–FTR–TRXs pathway, plant plastids harbor an NTR with a joint TRX domain, termed NTRC (Serrato et al., 2004), which uses NADPH to reduce the hydrogen peroxide scavenging enzyme 2-Cys peroxiredoxin (2-Cys PRX; Moon et al., 2006; Pérez-Ruiz et al., 2006; Alkhalfioui et al., 2007), showing that NADPH is also used in the chloroplast redox network (Spínola et al., 2008). Efforts to elucidate the role of NTRC in chloroplast performance, as well as its functional relationship with the classical FDX–FTR–TRX pathway, have led to a new model of chloroplast redox regulation according to which the redox balance of 2-Cys PRXs indirectly modulates TRX-regulated enzymes (Pérez-Ruiz et al., 2017). This model implies key roles for NADPH and hydrogen peroxide, as input and final sink of electrons, respectively. In this review, we update the current knowledge of the relationship of thiol-dependent redox regulation and antioxidant systems and discuss potential mechanisms of redox modulation of the chloroplast signaling function.

## NADPH and hydrogen peroxide at the crossroad of chloroplast thiol-dependent redox regulatory and antioxidant systems

**Figure 1:**
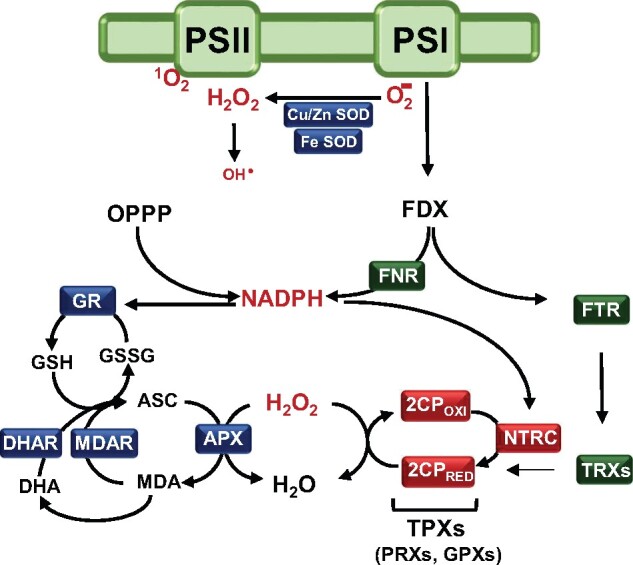
NADPH and hydrogen peroxide at the crossroad of chloroplast antioxidant and thiol-dependent redox regulatory systems. FDX, the final acceptor of the photosynthetic electron transport chain, fuels reducing equivalents to plastid thioredoxins (TRXs) with the participation of FTR. NADPH, which can be produced from sugars by the oxidative pentose phosphate pathway (OPPP), is also generated from FDX by the action of FDX–NADP reductase (FNR). Photosynthesis inevitably produces ROS including superoxide anion ($O_{2}^{-}$), which is converted to hydrogen peroxide (H_2_O_2_) by Cu/Zn- and Fe-dependent SODs. ASC provides reducing equivalents to scavenge H_2_O_2_ via ASC peroxidase (APX), producing oxidized intermediates MDA and DHA. Regeneration of ASC via MDAR and DHAR uses reduced GSH hence yielding oxidized GSH (GSSG), which is reduced back to GSH by an NADPH-dependent GR. Plastids harbor an additional group of hydrogen peroxide scavenging enzymes, TPXs, which include PRXs and GPXs (see Box 1). The scheme only represents the most abundant plastid TPX, 2-Cys PRX (2CP), which is predominantly reduced by NADPH-dependent NTRC, and, with lower efficiency (denoted by a thinner arrow), by TRXs.

In plant cells, chloroplasts constitute an important source of ROS (for details see Asada, 2006), which include production of singlet oxygen (^1^O_2_) at photosystem (PS) II and superoxide anion ($O_{2}^{-}$) at PSI (Figure 1). To avoid the oxidative damage of ROS and allow their signaling activity, chloroplasts harbor antioxidant systems, both enzymatic and non-enzymatic. Carotenoids, ascorbate (ASC), and glutathione (GSH) are the main non-enzymatic ROS scavenging systems (Pinnola and Bassi, 2018). Among the enzymatic systems, Cu/Zn- and Fe-superoxide dismutases (SODs) catalyze the conversion of superoxide anion to hydrogen peroxide (Pilon et al., 2011). The generation of hydrogen peroxide at PSI, with electrons derived from water oxidation at PSII, and the reduction of hydrogen peroxide to water constitutes the water–water cycle (Asada, 2000). Hydrogen peroxide, which can generate hydroxyl radicals (OH^•^) by the Fenton reaction (Khorobrykh et al., 2020), exerts an important signaling function and, thus, its concentration is tightly controlled by the action of ASC peroxidases (APXs) and thiol-dependent peroxidases (TPXs). APXs, of which plastids contain two isoforms, localized at the stroma (sAPX) and the thylakoid (tAPX), use ASC to reduce hydrogen peroxide yielding monodehydroascorbate (MDA), which can be further oxidized to dehydroascorbate (DHA; Maruta et al., 2016; Figure 1). ASC is regenerated by the action of MDA reductase (MDAR) and DHA reductase (DHAR) using GSH, which is thus oxidized (GSSG). Regeneration of GSH is catalyzed by NADPH-dependent GSH reductase (GR; Waszczak et al., 2018; Figure 1).

The other group of hydrogen peroxide scavenging enzymes, TPXs, include PRXs (Dietz, 2011) and GSH peroxidases (GPXs; Bela et al., 2015; Box 1). The activity of TPXs relies on the disulfide reductase activity of NTRC, TRXs, and glutaredoxins (GRXs; Collin et al., 2003; Navrot et al., 2006; Pérez-Ruiz et al., 2006; Couturier et al., 2011; Liebthal et al., 2018), thus, linking these hydrogen peroxide scavenging systems with the redox regulatory network of the chloroplast. GRXs, which are more specifically involved in protein deglutathionylation and iron–sulfur cluster incorporation, have important roles in plant development (Rouhier et al., 2015; Zaffagnini et al., 2019). As 2-Cys PRXs, the most abundant chloroplast TPX (Peltier et al., 2006), are preferentially reduced by NTRC, NADPH plays a relevant role supporting this hydrogen peroxide scavenging system (Figure 1). Therefore, NADPH, as the ultimate source of reducing power for all chloroplast enzymes involved in hydrogen peroxide scavenging, and hydrogen peroxide, as the sink of electrons, constitute functional links at the crossroad of chloroplast thiol-dependent redox regulatory network and antioxidant systems.

## Plant chloroplasts harbor a complex redox network

Redox regulation based on dithiol–disulfide interchange constitutes an essential regulatory mechanism that allows the rapid adaptation of chloroplast metabolism to light. Typical chloroplast TRXs include those of types *m* (four isoforms), *f* (two isoforms), *y* (two isoforms), *x*, and *z* (Geigenberger et al., 2017; Nikkanen and Rintamäki, 2019; Zaffagnini et al., 2019). Initial biochemical analyses identified the redox regulation of enzymes of the Calvin–Benson cycle (CBC) and showed the relevance of *f*-type TRXs in the light-dependent regulation of carbon fixation (Michelet et al., 2013). In addition, the identification of NADP malate dehydrogenase (NADP-MDH) as a target of *m*-type TRXs showed the redox regulation of chloroplast reducing power exchange through the malate valve (Selinski and Scheibe, 2019). Most plastid TRXs are able to reduce TPXs (Box 1), however, as TRXs *x* and *y* were shown to be more efficient reductants of 2-Cys PRXs (Collin et al., 2003, 2004), it was proposed an antioxidant function for these TRXs, in contrast with TRXs *f* and *m*, which were considered to function in redox regulation.

Box 1 Chloroplast TPXs

TPXs include PRXs and GPXs. In contrast with cofactor-based enzymatic antioxidant systems, TPXs catalyze the reduction of peroxides using the reducing equivalents of either one (1-Cys PRXs) or two (2-Cys PRXs) thiolic groups of cysteine residues at their active sites, termed peroxidatic (Cys_P_) and resolving (Cys_R_), the latter being absent in 1-Cys PRXs. The reaction mechanism of 2-Cys PRXs can be divided into three steps (Perkins et al., 2015): (i) the peroxide is attacked by Cys_P_, which becomes transiently oxidized to sulfenic acid, (ii) this intermediate reacts with Cys_R_ forming a disulfide and, when the peroxide is hydrogen peroxide, liberating a molecule of water, and (iii) the disulfide is reduced for a new catalytic cycle. 2-Cys PRXs are grouped as typical, homodimeric enzymes in which Cys_P_ and Cys_R_ are in different subunits, and atypical, monomeric enzymes with Cys_P_ and Cys_R_ in the same polypeptide. Atypical 2-Cys PRXs are classified as PRX Q and type II PRXs of which there are different isoforms: A, B, D, E, and F (Dietz, 2011). PRXs and GPXs are encoded by large gene families in higher plants. For example, Arabidopsis (*Arabidopsis thaliana*) contains 10 *PRX* (Dietz, 2011) and 8 *GPX* genes (Bela et al., 2015), which encode proteins targeted to different subcellular compartments. The chloroplast is by far the organelle with the largest content and types of TPXs. The Arabidopsis chloroplast contains two almost identical 2-Cys PRXs, termed A and B, PRX IIE, PRX Q, and two GPXs, GPX1 and GPX7. Regeneration of these TPXs is catalyzed by NTRC, TRXs, and TRX-like proteins, except Prx IIE, which is preferentially reduced by GRXs (Couturier et al., 2011), as summarized in the figure. Although most chloroplast TRXs are able to reduce 2-Cys PRXs, TRXs of the types *x* (Collin et al., 2003) and *y* (Collin et al., 2004) were identified as the most efficient reductants. Likewise, these TRX isoforms can reduce PRX Q (Collin et al., 2004), whereas GPXs receive reducing power from *y*-type TRXs (Navrot et al, 2006). NTRC, which is also able to reduce PRX Q (Liebthal et al., 2018), is the most efficient reductant of 2-Cys PRXs (Pulido et al., 2010). Based on their high abundance (Peltier et al., 2006) and high catalytic efficiency, 2-Cys PRXs have been considered to play a relevant antioxidant function in plant chloroplasts.

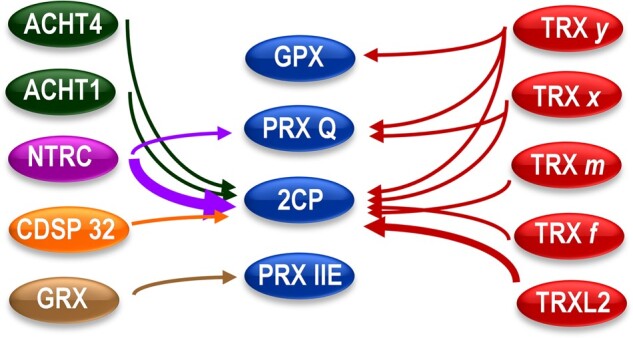

A search of genes encoding NTRs in plants identified the *NTRC* gene, which encodes a polypeptide containing both NTR and TRX domains. NTRC is unique to oxygenic photosynthetic organisms (Pascual et al., 2010; Nájera et al., 2017) and, in plants, shows localization in any type of plastid, though it is more abundant in chloroplasts (Serrato et al., 2004; Moon et al., 2006; Kirchsteiger et al., 2012). Truncated polypeptides containing either the NTR or the TRX domain of NTRC showed that this novel enzyme could display both activities (Serrato et al., 2004). The finding of 2-Cys PRX as target of NTRC helped to solve the actual biochemical properties of the enzyme, which is able to conjugate both NTR and TRX activities to efficiently reduce 2-Cys PRXs (Moon et al., 2006; Pérez-Ruiz et al., 2006; Alkhalfioui et al., 2007). Thus, NTRC could be considered as a TRX that bears its own NTR, which might explain the high catalytic efficiency of the enzyme. The catalytically active form of NTRC is a homodimer arranged in a head-to-tail conformation, which interacts with 2-Cys PRXs through the TRX domain (Pérez-Ruiz and Cejudo, 2009), and shows high affinity for NADPH (Bernal-Bayard et al., 2012). Additionally, NTRC could interact with plastid TRXs through its NTR domain; however, this activity of the enzyme remains controversial. Based on the finding that the overexpression of a NTRC variant with a dysfunctional TRX domain partially rescued the growth inhibition phenotype of the *ntrc* mutant, it was proposed that NTRC reduces TRXs, with *f*-type TRX being the most likely candidate (Toivola et al., 2013). Further supporting the NTR activity of NTRC, affinity chromatography trapping experiments identified TRX *z* as a putative target of the enzyme (Yoshida and Hisabori, 2016). However, it is not yet clear the ability of NTRC to reduce TRX *z* in vitro since the poor activity of NTRC as reductant of TRX *z* reported by Bohrer et al. (2012) is in contrast with the higher activity reported by Yoshida and Hisabori (2016).

The high efficiency of 2-Cys PRX reduction by NTRC supports the antioxidant function of this enzyme (Pérez-Ruiz et al., 2006). In line with this function, it was shown that Mg-protoporphyrin monomethyl ester cyclase, an enzyme of the chlorophyll biosynthesis pathway, is protected from oxidation by the NTRC-dependent activity of 2-Cys PRXs (Stenbaek et al., 2008). The redox state of 2-Cys PRXs is impaired in the Arabidopsis mutant devoid of NTRC, but not in the mutant devoid of TRX *x*, showing that NTRC is a more efficient reductant of 2-Cys PRXs in vivo than Trx *x* (Pulido et al., 2010), a notion confirmed by in vitro analysis (Yoshida and Hisabori, 2016). Interestingly, NTRC is unable to reduce PRX IIE and shows poor efficiency as a reductant of PRX Q (Yoshida and Hisabori, 2016). Although the capacity of NTRC to reduce GPXs remains to be elucidated, these results indicate that the antioxidant function of NTRC is mainly exerted through the hydrogen peroxide scavenging activity of 2-Cys PRXs, implying that NADPH acts as the ultimate source of reducing power supporting this antioxidant pathway.

## NTRC and 2-Cys PRXs modulate the activity of chloroplast TRXs

In recent years, the use of Arabidopsis mutants has led to a significant progress in the understanding of the functional relationship of the chloroplast redox regulated processes and antioxidant systems. As mentioned above, the FDX–FTR–TRXs pathway allows the light-dependent regulation of chloroplast metabolism (Figure 1). In support of the important function of this pathway, Arabidopsis mutants with decreased contents of the catalytic subunit of FTR (FTRc), hence with impaired fuel of electrons into the pathway, show severe chlorosis in leaf sectors near the petiole (Wang et al., 2014), similar to the phenotype of mutant plants with lower FTR activity (Hashida et al., 2018). In contrast, the deficiency of individual TRXs has low effect on growth phenotype indicating functional redundancy of the different plastid TRXs (Cejudo et al., 2019), except TRX *z* since mutants lacking this TRX show an albino phenotype (Arsova et al., 2010).

Unlike mutants defective in individual TRXs, the Arabidopsis mutant devoid of NTRC displays a severe growth retard phenotype, which is more pronounced under light limitation (Serrato et al., 2004; Lepistö et al., 2009). Moreover, the Arabidopsis *ntrc* mutant is highly sensitive to both biotic (Ishiga et al., 2012, 2016) and abiotic stress (Serrato et al., 2004; Chae et al., 2013). Since these stresses enhance ROS production, these findings are in line with the antioxidant function proposed for NTRC. However, the lack of NTRC also causes impairment of metabolic processes apparently unrelated with the antioxidant activity of the enzyme such as the redox regulation of ADP-glucose pyrophosphorylase (AGPase), a key regulatory enzyme of starch biosynthesis (Michalska et al., 2009; Lepistö et al., 2013). Similarly, NTRC participates in the redox regulation of chlorophyll biosynthesis enzymes such as Mg-protoporphyrin IX methyltransferase (CHLM), glutamyl-tRNA reductase (GluTR; Richter et al., 2013), and the CHLI subunit of Mg-chelatase (Pérez-Ruiz et al., 2014). Moreover, it was also shown that NTRC affects photoprotection mechanisms through non-photochemical quenching (NPQ; Thormählen et al., 2015; Carrillo et al., 2016; Naranjo et al., 2016).

Altogether, the above-mentioned results show the participation of NTRC in different processes previously known to be under the regulation of TRXs. Intriguingly, these processes are more severely impaired in the *ntrc* mutant than in plants lacking individual TRXs, hence raising the question of the functional relationship of NTRC with the FDX–FTR–TRXs redox system. This issue has been addressed by the analysis of Arabidopsis mutants combining the deficiencies of NTRC and individual TRXs. The simultaneous lack of NTRC and TRX *f*1 (Thormählen et al., 2015), TRXs *f*1 and *f*2, TRX *x* (Ojeda et al., 2017), or TRXs *m* (Da et al., 2017) causes severe aggravation of growth inhibition. Moreover, the light-dependent redox regulation of CBC enzymes was severely impaired in these mutants (Thormählen et al., 2015; Ojeda et al., 2017), suggesting that NTRC and the FDX–FTR–TRXs redox systems act concertedly. One possibility to explain this concerted action is that both redox systems share common targets. The interaction of NTRC and fructose bisphosphatase (FBPase; Nikkanen et al., 2016) lends support to this notion; however, in vitro assays showed the inability of NTRC to reduce FBPase (Yoshida and Hisabori, 2016; Ojeda et al., 2017), suggesting that the activity of NTRC is exerted without direct redox interaction with the targets.

**Figure 2:**
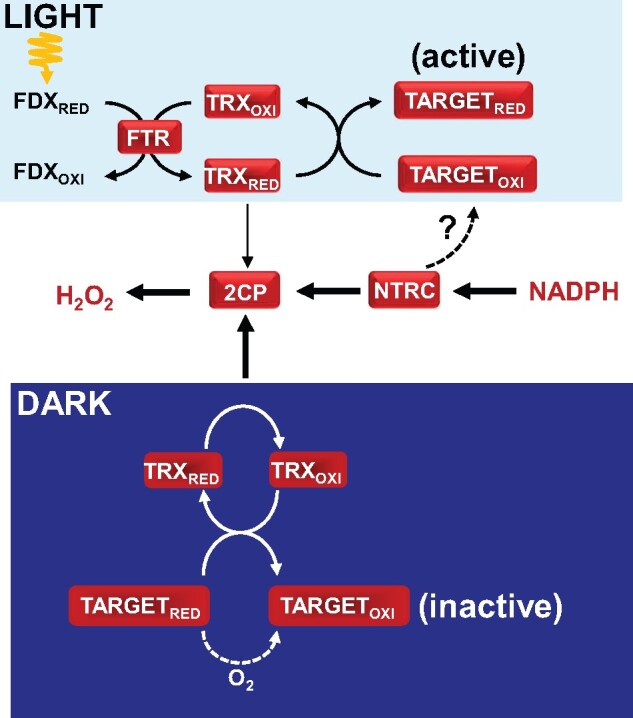
Chloroplast redox regulatory pathways in the light and the dark. The NTRC-2-Cys PRX (2CP) redox system constitutes a redox relay. During the day (LIGHT), the pool of TRXs is maintained reduced by photosynthetically reduced FDX. The redox state of 2-Cys PRXs is predominantly maintained by NTRC and, to a lesser extent, by TRXs (denoted by thick and thin arrows, respectively). Thus, the activity of NTRC avoids the drainage of electrons from TRXs, hence maintaining downstream targets reduced and active. Additionally, NTRC may directly interact with other chloroplast redox-regulated enzymes. During the night (DARK), input of reducing equivalents via reduced FDX ceases and TRXs mediate the transfer of reducing equivalents from reduced targets to 2-Cys PRXs and hydrogen peroxide, while targets consequently become oxidized and inactive. Reoxidation of redox regulated enzymes by molecular oxygen may also occur in the aerobic environment of the chloroplast.

The question arising is thus the mechanism of action of NTRC. Intriguingly, Arabidopsis mutants with decreased contents of 2-Cys PRXs in the *ntrc* background recovered a growth phenotype similar to the wild type, indicating that decreased contents of 2-Cys PRXs suppress the *ntrc* phenotype. Furthermore, the overexpression of 2-Cys PRXs, which has no significant effect in wild-type plants, resulted in further growth impairment in the *ntrc* background (Pérez-Ruiz et al., 2017), showing that the severity of the *ntrc* phenotype depends on 2-Cys PRXs levels. Based on these results, it was proposed that NTRC maintains the redox state of 2-Cys PRXs, thus avoiding drainage of electrons from the pool of TRXs and, consequently, maintaining their targets reduced, hence active during the day (Figure 2). The accumulation of oxidized 2-Cys PRXs in the absence of NTRC would increase drainage of reducing equivalents from the pool of TRXs impairing the redox regulation of TRX-dependent processes. Further supporting the role of 2-Cys PRXs in chloroplast redox homeostasis, the very severe growth inhibition phenotypes of mutants combining the deficiencies of NTRC and TRXs *f* or *x* were also rescued by decreasing the contents of 2-Cys PRXs (Pérez-Ruiz et al., 2017; Ojeda et al., 2018a). This model of chloroplast redox regulation implies that NTRC, by controlling the redox balance of 2-Cys PRXs, affects the redox regulation of any TRX-dependent process in the organelle, providing an explanation for the fact that the activity of this enzyme affects such a variety of processes yet with no direct interaction with the TRX-regulated targets. However, as mentioned above, NTRC interacts with different chloroplast enzymes; thus, the question of whether NTRC acts by directly regulating specific enzymes remains an open issue (Figure 2).

Initial studies of light-dependent regulation of chloroplast metabolism established the reductive activation of enzymes in the light, and also their rapid oxidative inactivation in the dark. While most efforts have been devoted to identifying the mechanism of reductive activation, the mechanism of oxidation has remained poorly understood. Based on the rapid inactivation of FBPase upon oxygen addition to chloroplasts kept under anaerobic conditions, it was proposed that the participation of oxygen in the process of enzyme inactivation occurs either directly or through the formation of superoxide and hydrogen peroxide (Leegood and Walker, 1980). Accordingly, Knuesting and Scheibe (2018) proposed that light-dark regulation of chloroplast enzymes would be operated by a redox switch; in the aerobic environment of the chloroplast, redox-sensitive enzymes are continuously oxidized by oxygen and re-reduced by reducing equivalents provided by the photosynthetic electron transport chain in the light, with the enzymes remaining oxidized in the dark when the electron flow stops. In contrast, Schürmann and Buchanan (2008) proposed the participation of TRXs, which would transfer reducing equivalents either directly to oxygen or, via PRX, to hydrogen peroxide, however, experimental evidence in support of the participation of these enzymes in the process of oxidation was lacking. The participation of 2-Cys PRXs in the maintenance of chloroplast redox homeostasis suggested their involvement in the process of enzyme oxidation, a notion confirmed by the recent finding that Arabidopsis mutants deficient in the two chloroplast 2-Cys PRXs (A and B) display delayed enzyme oxidation in the dark (Ojeda et al., 2018b; Vaseghi et al., 2018; Yoshida et al., 2018; Figure 2). It is known that 2-Cys PRXs interact with different proteins in the chloroplast stroma (Cerveau et al., 2016a; Liebthal et al., 2020) and, thus, could act as an oxidant relay by direct interaction with these targets, as proposed in mammalian cells (Stocker et al., 2017). However, in vitro experiments showed that oxidation of chloroplast enzymes is mediated by TRXs (Ojeda et al., 2018b; Vaseghi et al., 2018), with TRX-like2 (TRX L2) showing higher efficiency (Yoshida et al., 2018). Therefore, these data support the notion that 2-Cys PRXs transfer reducing equivalents to hydrogen peroxide, which thus acts as a final sink of electrons from reduced enzymes in the dark. It should be emphasized that the two models, the participation of oxygen and the participation of 2-Cys PRXs, are not mutually exclusive. Indeed, albeit delayed, dark-triggered enzyme oxidation still occurs in mutant plants lacking 2-Cys PRXs, which indicates the participation of additional mechanism(s). Furthermore, specific metabolites such as substrates and products of stromal reactions influence redox interconversions of chloroplast enzymes (reviewed in Knuesting and Scheibe, 2018), highlighting the molecular complexity of thiol–disulfide interchange in the organelle.

The participation of 2-Cys PRXs in chloroplast redox regulation (Figure 2) has important implications. First, hydrogen peroxide has the key function of acting as a sink for reducing equivalents from redox regulated enzymes; moreover, since NADPH is the source of reducing power for NTRC, the NADPH/NADP^+^ ratio would be an important signal determining the rate of biosynthetic pathways of the organelle. In this regard, the level of NADPH increases upon illumination and rapidly decreases in the dark (Lim et al., 2020). Previously, it was proposed that there exists a photosynthetic control that allows the coordination of photochemical reactions, synthesis of ATP and NADPH, and the operation of chloroplast metabolic pathways in order to optimize the use of excitation energy at low irradiance and to dissipate the excess energy at high irradiance (Foyer et al., 1990). The water–water cycle, which consumes electrons in production and further reduction of hydrogen peroxide (Asada, 2000), has long been considered as a mechanism to dissipate excess reducing equivalents, though it was found not to be relevant when CO_2_ assimilation is restricted (Driever and Baker, 2011). The transfer of reducing equivalents to hydrogen peroxide catalyzed by 2-Cys PRXs, which accelerates the oxidation of stromal enzymes in the dark, may serve as an additional mechanism to dissipate excess reducing equivalents under high irradiance.

Furthermore, it is well established that chloroplast 2-Cys PRXs, like their counterparts in other eukaryotes (Wood et al., 2003; Perkins et al., 2015), undergo overoxidation, which causes the inactivation of the peroxidase activity of the enzyme (Kirchsteiger et al., 2009; Puerto-Galán et al., 2013). It was found that 2-Cys PRXs overoxidation shows circadian oscillation and based on these results, it was proposed that redox cycles of 2-Cys PRXs constitute a universal marker for circadian rhythms (Edgar et al., 2012). In plant chloroplasts, NTRC, which affects the level of 2-Cys PRXs overoxidation (Puerto-Galán et al., 2015), might affect endogenous circadian rhythms (Box 2), but more work is needed to establish the role of NTRC and 2-Cys PRXs in the circadian clock.

Box 2 2-Cys PRX overoxidation and the circadian clock

The catalytic cycle of 2-Cys PRXs correlates with two stable conformations of the enzyme, fully folded (FF) and locally unfolded (LU; see the figure). The attack of the peroxide by the thiolate group of the peroxidatic cysteine (Cys_P_), which forms the sulfenic intermediate, occurs in the FF conformation. The enzyme must undergo a conformational change to the LU conformation to allow the reaction of the sulfenic intermediate with the resolving cysteine (Cys_R_), which results in the formation of a disulfide bond (Wood et al., 2003). 2-Cys PRXs from eukaryotes contain two well-conserved motifs, GGLG and YF, which bury apart Cys_P_ and Cys_R_, stabilizing the FF conformation and favoring overoxidation of the sulfenic intermediate to sulfinic acid under oxidizing conditions. Overoxidation, which can be recovered by the action of sulfiredoxin (SRX), has deep effects on the conformation and catalytic activity of 2-Cys PRXs. The overoxidized enzyme forms aggregates that lack peroxidase activity and display chaperone function (Puerto-Galán et al., 2013), hence allowing the local accumulation of hydrogen peroxide, which can then exert signaling function according to the floodgate hypothesis (Wood et al., 2003). Moreover, overoxidation of 2-Cys PRX from different organisms, including plants, shows circadian oscillation leading to the proposal that these enzymes are universal markers of metabolic circadian rhythms (Edgar et al., 2012; see the figure). Remarkably, most prokaryotic 2-Cys PRXs lack the GGLC and YF motifs being thus insensitive to overoxidation; therefore, this post-translational modification is considered a gain-of-function of eukaryotic 2-Cys PRXs that favor the signaling activity of hydrogen peroxide in these organisms. Nevertheless, 2-Cys PRX from photosynthetic prokaryotes, such as the filamentous cyanobacteria *Anabaena*, is sensitive to overoxidation (Pascual et al., 2010), indicating that filamentous cyanobacteria, considered the ancestors of present chloroplasts, have a eukaryotic-type strategy to cope with hydrogen peroxide. Similarly, chloroplast 2-Cys PRXs are sensitive to overoxidation (Kirchsteiger et al., 2009), which peaks in the early morning after the night–day transition (Edgar et al., 2012; Cerveau et al., 2016b), further supporting the relevant signaling activity of hydrogen peroxide produced in the organelle. Interestingly, Arabidopsis mutants lacking NTRC show severely diminished 2-Cys PRXs overoxidation (Puerto-Galán et al., 2015), indicating the participation of NTRC in the control of 2-Cys PRX overoxidation and, consequently, in the activity of these enzymes as markers of metabolic rhythms. These results suggest a functional relationship of the redox state of the chloroplast and the circadian clock, an issue that deserves future attention.

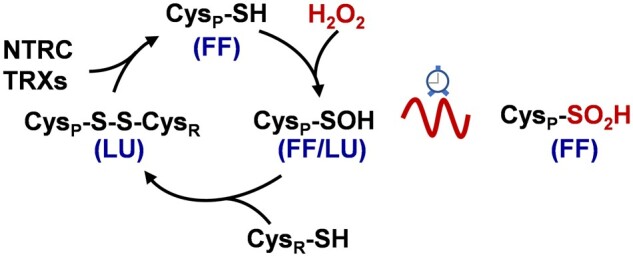

Intriguingly, the lack of 2-Cys Prxs causes only a modest inhibition of growth at the adult stage, at least under standard growth conditions (Awad et al., 2015; Ojeda et al., 2018b). It is worth emphasizing that plant chloroplasts are unique systems concerning redox regulation because the redox state of these organelles is rapidly adjusted in response to changes in light intensity, which in nature occur continuously and unpredictably. The Arabidopsis mutant devoid of 2-Cys PRXs shows higher sensitivity than wild-type plants to high light and oxidative stress (Awad et al., 2015), however, this mutant shows better growth performance than the wild type when grown under short fluctuating light pulses, which was attributed to the inefficient inactivation of redox-regulated chloroplast enzymes in the absence of 2-Cys PRXs (Vaseghi et al., 2018). In line with these observations, the Arabidopsis mutant lacking GPX7 displays better growth of rosette leaves and higher density of lateral roots than the wild type (Passaia et al., 2014). The better growth of mutants lacking 2-Cys PRXs, in short fluctuating light pulses, and GPX7 suggests that the imbalance of the redox-regulated enzymes, which would be more reduced and, thus, more active in these mutants, may have beneficial effects of plant growth.

## Redox modulation of chloroplast sensor activity

Except for a small number of proteins encoded by the chloroplast genetic information, most of the proteins of the organelle are encoded by the nuclear genome. Thus, the transcriptional activity of both genomes must be tightly coordinated via a bi-directional communication, from nucleus-to-chloroplast (anterograde signaling), and from chloroplast-to-nucleus (retrograde signaling). Retrograde signals informing of the chloroplast state are operative in the process of chloroplast biogenesis (biogenic control) and in mature chloroplasts in response to environmental cues (operational control; Chan et al., 2016a). Therefore, besides the primary function of chloroplasts as a source of metabolic intermediates, these organelles have an important signaling activity as sensors of environmental conditions. As discussed above, the redox state of chloroplast enzymes reflects the metabolic performance of the organelle. In this section, we discuss possible mechanisms of redox regulation of chloroplast sensor activity.

**Figure 3:**
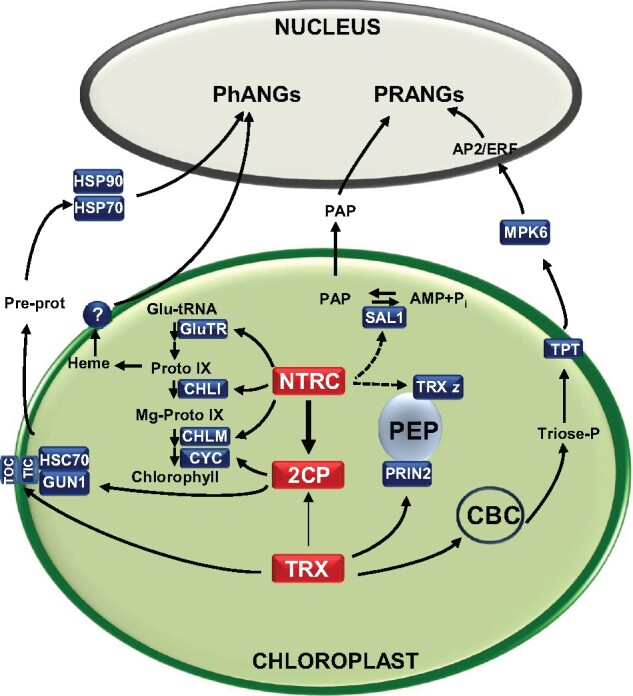
The chloroplast redox network may modulate retrograde signaling through several potential mechanisms. Retrograde signaling coordinates the activity of nuclear and plastid genomes during the process of chloroplast biogenesis (biogenic control), affecting the expression of PhANGs, and in mature chloroplasts in response to environmental cues (operational control), affecting PRANGs. The redox state of the chloroplast may constitute an additional layer of regulation that modulates biogenic and operational retrograde signaling. The NTRC–2-Cys PRX redox system controls tetrapyrrole biosynthesis enzymes such as GluTR, Mg-protoporphyrin IX methyltransferase (CHLM), the CHLI subunit of Mg-chelatase, and the Mg-protoporphyrin IX methylester cyclase (CYC). Biogenic retrograde signaling may thus be affected by the redox control of tetrapyrrole metabolism, as heme synthesis in the chloroplast promotes the expression of PhANGs. Import of pre-proteins (Pre-prot) may be affected by redox modifications of components of the translocons at the outer (TOC) and inner (TIC) chloroplast membranes or the interaction of 2-Cys PRX (2CP) with the GUN1–HSC70 complex, which triggers retrograde signaling mediated by cytosolic chaperones HSP90 and HSP70. Redox regulation of PEP-dependent transcription may be exerted by NTRC-dependent regulation of TRX *z* and PRIN2, which can also be regulated by TRXs. The redox state of phosphatase SAL1, which affects the levels of operational retrograde signal PAP, might be influenced by the NTRC–2-Cys PRX system. Operational retrograde signaling in response to high light mediated by kinase MPK6 and transcription factors APETALA2/ETHYLENE RESPONSE FACTOR (AP2/ERF) might be modulated by TRXs that regulate the Calvin–Benson cycle (CBC), the source of triose phosphate, which is translocated from the chloroplast via a TPT. Dashed arrows indicate indirect or putative interactions.

Mature chloroplasts emit a variety of signaling molecules that act as operational retrograde signals affecting the expression of plastid redox-associated nuclear genes (PRANGs) in response to environmental cues (Chan et al., 2016a). One such signaling molecule is 3′-phosphoadenosine-5′-phosphate (PAP), the concentration of which increases in response to abiotic stresses such as drought and high light; hence, it was proposed to function as an operational retrograde signal (Estavillo et al., 2011). PAP concentration is maintained by the SAL1 phosphatase, a redox-regulated enzyme that catalyzes the dephosphorylation of PAP to AMP and Pi. Interestingly, the *ntrc* mutant shows lower SAL1 activity than the wild type in response to high light (Chan et al., 2016b), suggesting a function for NTRC in the redox regulation of this enzyme. Although the molecular basis of this effect of NTRC on SAL1 activity remains to be elucidated, it may be one of the targets connecting the operational retrograde signaling with the redox state of the chloroplast (Figure 3). An additional operational retrograde signaling pathway in response to high light involves the translocation of triose phosphate from the chloroplast via a triose phosphate translocator (TPT), the activation of kinase MPK6 and transcription factors APETALA2/ETHYLENE RESPONSE FACTOR (AP2/ERF; Vogel et al., 2014). As stated above, the CBC, which is the source of triose phosphate, is under redox regulation which, thus, may be an additional layer of modulation of the TPT/MPK6 operational retrograde signaling pathway (Figure 3).

Biogenic retrograde signals coordinate multiple processes leading to the differentiation of a functional chloroplast at early stages of plant development. Interestingly, Arabidopsis mutants deficient in components of the chloroplast redox regulatory network show defective seedling development. This is the case of mutants lacking NTRC and TRXs *f*, which show defective chloroplast ultrastructure in cotyledons and high mortality at the seedling stage (Ojeda et al., 2017), suggesting that plastid redox regulation may also modulate biogenic retrograde signaling. Chloroplast biogenesis requires the activation of the transcriptional machinery of the organelle, which is performed by two RNA polymerases, nuclear-encoded (NEP) and plastid-encoded (PEP; Jarvis and López-Juez, 2013). Full PEP functionality depends on the correct assembly of a multi-protein complex formed by the PEP core subunits, sigma factors, and PEP-associated proteins (PAPs), which include TRX *z* (Pfannschmidt et al., 2015). Unlike the other plastidial TRXs, Arabidopsis mutants lacking TRX *z* develop albino cotyledons due to defects in chloroplast biogenesis (Arsova et al., 2010), which is in line with the seedling lethal phenotype of mutant plants deficient in other PAPs (Pfannschmidt et al., 2015). In principle, the presence of TRX *z* as part of the PEP complex suggested the redox control of chloroplast gene expression, however, the finding that the phenotype of the *trxz* mutant could be restored by redox-insensitive variants of TRX *z* pointed to a minor role of the redox activity of the enzyme in PEP-dependent transcription (Wimmelbacher and Börnke, 2014). Nevertheless, the redox regulation of PEP transcription and, thus, of chloroplast biogenesis, via TRX *z* remains as a likely possibility, which deserves future attention (Figure 3). Another candidate target of the redox regulation of plastid transcription is plastid redox insensitive 2 (PRIN2), which is essential for full PEP activity (Kindgren et al., 2012; Hernández-Verdeja et al., 2020). PRIN2 undergoes redox regulation by the interconversion of a dimeric (inactive) form, linked by a disulfide bridge, and a monomeric (active) form produced upon light-triggered reduction of the disulfide (Díaz et al., 2018). Although TRX *z* and TRX *f*1 reduce PRIN2 in vitro, it is not yet clear whether these TRXs participate in the process. Based on the notion that the NTRC-2-Cys PRX redox system controls the reducing capacity of the pool of plastid TRXs (Pérez-Ruiz et al., 2017), the redox state of PRIN2 and, thus, the activity of the PEP machinery may also by affected by this redox system (Figure 3).

Disruption of chloroplast development at the early seedling stage by treatment with the carotenoid synthesis inhibitor norflurazon (NF) or the plastid translation inhibitor lincomycin provokes the repression of photosynthesis-associated nuclear genes (PhANGs). These inhibitors were used to isolate the *genomes uncoupled* (*gun*) mutants, defective in biogenic retrograde signaling (Susek et al., 1993). Five of the six *gun* mutants identified (*gun2-6*) are defective in the metabolism of tetrapyrroles, chlorophyll, and heme (Larkin, 2016). Several steps of the tetrapyrrole biosynthesis pathway are redox regulated (Brzezowski et al., 2015), hence, the levels of intermediates of the pathway, and their function as retrograde signals, may be modulated by the redox state of the chloroplast. The first specific reaction of the chlorophyll branch of the pathway, the insertion of Mg^2+^ into protoporphyrin IX, is catalyzed by Mg-chelatase, a heterotrimeric enzyme composed of subunits CHLD, CHLH, and CHLI (Tanaka et al., 2011). The finding that the *gun5* mutant is defective in the CHLH subunit (Mochizuki et al., 2001), together with the regulatory role of GUN4 on CHLH (Larkin et al., 2003; Peter and Grimm, 2009) uncovered the key role of this complex in retrograde signaling. NTRC affects the redox regulation of CHLI (Pérez-Ruiz et al., 2014), GluTR, and CHLM (Richter et al., 2013), whereas 2-Cys PRX protects Mg-protoporphyrin monomethyl ester cyclase (Stenbaek et al., 2008). These results show that the NTRC–2-Cys PRXs redox system controls the stability of tetrapyrrole biosynthesis enzymes (Richter et al., 2018) and, therefore, may modulate biogenic retrograde signaling (Figure 3).

Of the *GUN* genes initially identified, the function of *GUN1* has remained elusive. Recently, immunoprecipitation experiments in de-etiolating seedlings showed that GUN1 interacts with cpHSC70-1, a chloroplast chaperone that participates in protein import into the organelle. Moreover, in the *gun1* mutant or in chemically damaged plastids, the accumulation of unimported preproteins in the cytosol acts as a retrograde signal leading to the induction of PhANGs via the accumulation of the cytosolic chaperone HSP90 (Wu et al., 2019). These relevant findings led to the proposal that GUN1 functions in protein import during chloroplast biogenesis, uncovering that folding stress in the cytosol triggers a retrograde signal (Wu et al., 2019). Interestingly, together with cpHSC70, 2-Cys PRX A was identified among the candidate partners of GUN1 during early chloroplast biogenesis (Wu et al., 2019), suggesting that redox signals may affect import-related retrograde communication. This possibility is compatible with the observation that the import of several photosynthetic preproteins is affected by the redox status of the organelle (Balsera et al., 2010). Moreover, a different study found that both 2-Cys PRX isoforms, A and B, were among the GUN1-interactors identified by coimmunoprecipitation experiments performed in adult leaves, which include chloroplast factors involved in protein synthesis and homeostasis (Tadini et al., 2016). Although the participation of 2-Cys PRX in retrograde signaling is unclear, its interaction with GUN1 during chloroplast biogenesis and in rosette leaves suggests that these enzymes may contribute to both biogenic and operational control of chloroplast function. In support of this possibility, cpHSC70 was identified in protein complexes, which also contain 2-Cys PRXs, purified from Arabidopsis plants expressing a tagged version of NTRC (González et al., 2019). Altogether, these findings suggest that protein homeostasis and, thus, retrograde signaling might be under redox regulation via the interaction of NTRC and/or 2-Cys PRX with the GUN1–cpHSC70 complex (Figure 3).

## Concluding remarks

By their sessile lifestyle, plants must cope with continuous and unpredictable changes of environmental conditions, this probably being the reason for the complex redox network of plant chloroplasts. The FDX–FTR–TRXs redox pathway, discovered about 50 years ago (recently reviewed by Buchanan, 2016), links the regulation of photosynthetic metabolism to light in contrast to redox regulation in non-photosynthetic organisms, which rely on NADPH. However, the discovery of NTRC, a plastid-localized enzyme with high affinity for NADPH, showed that the chloroplast redox network also uses NADPH. Intriguingly, the deficiency of NTRC has severe phenotypic effects, in contrast with the minor effects caused by the deficiency of individual plastid TRXs, which indicates a central function of the NADPH-dependent redox system in chloroplast performance. Genetic analyses led to the proposal that NTRC and the FDX–FTR–TRXs pathway are integrated by the redox balance of 2-Cys PRXs. The role of 2-Cys PRXs modulating chloroplast redox homeostasis was further extended by the participation of these enzymes in the oxidative inactivation of chloroplast enzymes in the dark. The redox balance of 2-Cys PRXs is maintained by NTRC, the most relevant reductant of the enzyme, avoiding drainage of reducing equivalents from the pool of TRXs, which allows the activation of downstream TRX targets during the day and its oxidative inactivation during the night. This model, however, does not exclude the previously proposed role of molecular oxygen in the oxidation of redox-regulated enzymes in the aerobic environment of the organelle. The functional relationship of thiol-dependent antioxidant systems and the redox regulatory network of the chloroplast raises several questions that should be addressed in the future (see the “Outstanding Questions”). A central issue to advance in the understanding of chloroplast redox regulation is the use of sensor probes for accurately determining the in vivo concentrations of NADPH and hydrogen peroxide. An additional aspect to be taken into consideration is that the NTRC–2-Cys PRXs system oxidizes NADPH to reduce hydrogen peroxide in vitro. Thus, to avoid futile oxidation of NADPH in vivo, the activity of the NTRC–2-Cys PRXs system may be tightly controlled. It is well established that chloroplast 2-Cys PRXs undergo overoxidation, which might affect endogenous circadian rhythms. This mechanism and/or additional post-translational modifications may meet the regulatory requirements of the system, but more work is needed to establish the biochemical regulation of the NTRC–2-Cys PRXs redox system. Finally, chloroplasts have important functions as sensors of environmental conditions and as a source of retrograde signals that coordinate the growth of plants at different developmental stages. Chloroplast redox state might be an additional layer of regulation to modulate the sensor activity of the organelle, thus the connection between redox transmitters and signaling pathways is an issue that deserves further exploration.

**Outstanding questions**

- How is the NTRC–2-Cys PRX redox system regulated to avoid the futile loss of NADPH in vivo?
- What is the interplay between enzymatic and non-enzymatic mechanisms governing the redox state of chloroplast enzymes?
- What is the physiological relevance of circadian oscillation of 2-Cys PRXs overoxidation?
- How do the dynamic changes of NADPH and H_2_O_2_ affect chloroplast performance?
- Which additional mechanism(s), beside 2-Cys PRXs, participate in the process of chloroplast enzyme oxidation in the dark?
- What is the role of individual chloroplast TRXs in enzyme oxidation in the dark?
- How do chloroplast redox signals modulate biogenic and operational retrograde signaling pathways?

## Notes

F.J.C., M.-C.G., and J.M.P.-R. conceived the contents and wrote the manuscript. Author for contact ensures that all authors agree to the list of authors and the identified contribution of those authors.

The author responsible for distribution of materials integral to the findings presented in this article in accordance with the policy described in the Instructions for Authors (https://academic.oup.com/plphys/pages/general-instructions) is: Francisco Javier Cejudo (fjcejudo@us.es).

## References

[kiaa062-B1] Alkhalfioui F , Renard M, Montrichard F (2007) Unique properties of NADP-thioredoxin reductase C in legumes. J Exp Bot 58**:** 969–978 17185738 10.1093/jxb/erl248

[kiaa062-B2] Arsova B , Hoja U, Wimmelbacher M, Greiner E, Ustun S, Melzer M, Petersen K, Lein W, Börnke F (2010) Plastidial thioredoxin *z* interacts with two fructokinase-like proteins in a thiol-dependent manner: evidence for an essential role in chloroplast development in Arabidopsis and *Nicotiana benthamiana*. Plant Cell 22**:** 1498–1515 20511297 10.1105/tpc.109.071001 PMC2899873

[kiaa062-B3] Asada K (2000) The water–water cycle as alternative photon and electron sinks. Phil Trans R Soc Lond B 355**:** 1419–1431 11127996 10.1098/rstb.2000.0703 PMC1692883

[kiaa062-B4] Asada K (2006) Production and scavenging of reactive oxygen species in chloroplasts and their functions. Plant Physiol 141**:** 391–396 16760493 10.1104/pp.106.082040 PMC1475469

[kiaa062-B5] Awad J , Stotz HU, Fekete A, Krischke M, Engert C, Havaux M, Berger S, Mueller MJ (2015) 2-Cysteine peroxiredoxins and thylakoid ascorbate peroxidase create a water–water cycle that is essential to protect the photosynthetic apparatus under high light stress conditions. Plant Physiol 167**:** 1592–1603 25667319 10.1104/pp.114.255356 PMC4378167

[kiaa062-B6] Balsera M , Buchanan BB (2019) Evolution of the thioredoxin system as a step enabling adaptation to oxidative stress. Free Radic Biol Med 140**:** 28–35 30862542 10.1016/j.freeradbiomed.2019.03.003

[kiaa062-B7] Balsera M , Soll J, Buchanan BB (2010) Redox extends its regulatory reach to chloroplast protein import. Trends Plant Sci 15**:** 515–521 20688558 10.1016/j.tplants.2010.06.002

[kiaa062-B8] Bela K , Horvatz E, Gallé A, Szabados L, Tari I, Csiszár J (2015) Plant glutathione peroxidases: emerging roles of the antioxidant enzymes in plant development and stress responses. J Plant Physiol 176**:** 192–201 25638402 10.1016/j.jplph.2014.12.014

[kiaa062-B9] Bernal-Bayard P , Hervás M, Cejudo FJ, Navarro JA (2012) Electron transfer pathways and dynamics of chloroplast NADPH-dependent thioredoxin reductase C (NTRC). J Biol Chem 287**:** 33865–33872 22833674 10.1074/jbc.M112.388991 PMC3460481

[kiaa062-B10] Bohrer AS , Massot V, Innocenti G, Reichheld JP, Issakidis-Bourguet E, Vanacker H (2012) New insights into the reduction systems of plastidial thioredoxins point out the unique properties of thioredoxin *z* from Arabidopsis. J Exp Bot 63**:** 6315–6323 23096001 10.1093/jxb/ers283

[kiaa062-B11] Brzezowski P , Richter AS, Grimm B (2015) Regulation and function of tetrapyrrole biosynthesis in plants and algae. Biochim Biophys Acta 1847**:** 968–985 25979235 10.1016/j.bbabio.2015.05.007

[kiaa062-B12] Buchanan BB (2016) The path to thioredoxin and redox regulation in chloroplasts. Annu Rev Plant Biol 67**:** 1–24 27128465 10.1146/annurev-arplant-043015-111949

[kiaa062-B13] Carrillo LR , Froehlich JE, Cruz JA, Savage L, Kramer DM (2016) Multi-level regulation of the chloroplast ATP synthase: the chloroplast NADPH thioredoxin reductase C (NTRC) is required for redox modulation specifically under low irradiance. Plant J 87**:** 654–663 27233821 10.1111/tpj.13226

[kiaa062-B14] Cejudo FJ , Ojeda V, Delgado-Requerey V, González M, Pérez-Ruiz JM (2019) Chloroplast redox regulatory mechanisms in plant adaptation to light and darkness. Front Plant Sci 10**:** 380 31019520 10.3389/fpls.2019.00380 PMC6458286

[kiaa062-B15] Cerveau D , Kraut A, Stotz HU, Mueller MJ, Couté Y, Rey P (2016a) Characterization of the *Arabidopsis thaliana* 2-Cys peroxiredoxin interactome. Plant Sci 252**:** 30–41 27717466 10.1016/j.plantsci.2016.07.003

[kiaa062-B16] Cerveau D , Ouahrani D, Marok MA, Blanchard L, Rey P (2016b) Physiological relevance of plant 2-Cys peroxiredoxin overoxidation level and oligomerization status. Plant Cell Environ 39**:** 103–119 26138759 10.1111/pce.12596

[kiaa062-B17] Chae HB , Moon JC, Shin MR, Chi YH, Jung YJ, Lee SY, Nawkar GM, Jung HS, Hyun JK, Kim WY, et al (2013) Thioredoxin reductase type C (NTRC) orchestrates enhanced thermotolerance to Arabidopsis by its redox-dependent holdase chaperone function. Mol Plant 6**:** 323–336 23024205 10.1093/mp/sss105

[kiaa062-B18] Chan KX , Phua SY, Crisp P, McQuinn R, Pogson BJ (2016a) Learning the languages of the chloroplast: retrograde signaling and beyond. Annu Rev Plant Biol 67**:** 25–53 26735063 10.1146/annurev-arplant-043015-111854

[kiaa062-B19] Chan KX , Mabbitt PD, Phua SY, Mueller JW, Nisar N, Gigolashvili T, Stroeher E, Grassl J, Arlt W, Estavillo GM, et al (2016b) Sensing and signaling of oxidative stress in chloroplasts by inactivation of the SAL1 phosphoadenosine phosphatase. Proc Natl Acad Sci U S A 113**:** E4567–E4576 27432987 10.1073/pnas.1604936113 PMC4978270

[kiaa062-B20] Collin V , Issakidis-Bourguet E, Marchand C, Hirasawa M, Lancelin JM, Knaff DB, Miginiac-Maslow M (2003) The Arabidopsis plastidial thioredoxins: new functions and new insights into specificity. J Biol Chem 278**:** 23747–23752 12707279 10.1074/jbc.M302077200

[kiaa062-B21] Collin V , Lamkemeyer P, Miginiac-Maslow M, Hirasawa M, Knaff DB, Dietz KJ, Issakidis-Bourguet E (2004) Characterization of plastidial thioredoxins from Arabidopsis belonging to the new *y*-type. Plant Physiol 136**:** 4088–4095 15531707 10.1104/pp.104.052233 PMC535839

[kiaa062-B22] Couturier J , Ströher E, , Albetel A-N, Roret T, Muthuramalingam M, Tarrago L, Seidel T, Tsan P, Jacquot J-P, et al (2011) Arabidopsis chloroplastic glutaredoxin C5 as a model to explore molecular determinants for iron-sulfur cluster binding into glutaredoxins. J Biol Chem 286**:** 27515–27527 21632542 10.1074/jbc.M111.228726 PMC3149344

[kiaa062-B23] Da Q , Wang P, Wang M, Sun T, Jin H, Liu B, Wang J, Grimm B, Wang HB (2017) Thioredoxin and NADPH-dependent thioredoxin reductase C regulation of tetrapyrrole biosynthesis. Plant Physiol 175**:** 652–666 28827456 10.1104/pp.16.01500 PMC5619880

[kiaa062-B24] Díaz MG , Hernández-Verdeja T, Kremnev D, Crawford T, Dubreuil C, Strand A (2018) Redox regulation of PEP activity during seedling establishment in *Arabidopsis thaliana*. Nat Commun 9**:** 50 29298981 10.1038/s41467-017-02468-2 PMC5752674

[kiaa062-B25] Dietz KJ (2011) Peroxiredoxins in plants and cyanobacteria. Antioxid Redox Signal 15**:** 1129–1159 21194355 10.1089/ars.2010.3657 PMC3135184

[kiaa062-B26] Driever SM , Baker NR (2011) The water–water cycle in leaves is not a major alternative electron sink for dissipation of excess excitation energy when CO_2_ assimilation is restricted. Plant Cell Environ 34**:** 837–846 21332508 10.1111/j.1365-3040.2011.02288.x

[kiaa062-B27] Edgar RS , Green EW, Zhao Y, van Ooijen G, Olmedo M, Qin X, Xu Y, Pan M, Valekunja UK, Feeney KA, et al (2012) Peroxiredoxins are conserved markers of circadian rhythms. Nature 485**:** 459–464 22622569 10.1038/nature11088 PMC3398137

[kiaa062-B28] Estavillo GM , Crisp PA, Pornsiriwong W, Wirtz M, Collinge D, Carrie C, Giraud E, Whelan J, David P, Javot H, et al (2011) Evidence for a SAL1-PAP chloroplast retrograde pathway that functions in drought and high light signaling in Arabidopsis. Plant Cell 23**:** 3992–4012 22128124 10.1105/tpc.111.091033 PMC3246320

[kiaa062-B29] Ferrer-Sueta G , Manta B, Botti H, Radi R, Trujillo M, Denicola A (2011) Factors affecting protein thiol reactivity and specificity in peroxide reduction. Chem Res Toxicol 24**:** 434–450 21391663 10.1021/tx100413v

[kiaa062-B30] Foyer, Furbank R , Harbinson J, Horton P (1990) The mechanisms contributing to photosynthetic control of electron transport by carbon assimilation in leaves. Photosynth Res 25**:** 83–100 24420275 10.1007/BF00035457

[kiaa062-B31] Geigenberger P , Thormählen I, Daloso DM, Fernie AR (2017) The unprecedented versatility of the plant thioredoxin system. Trends Plant Sci 22**:** 249–262 28139457 10.1016/j.tplants.2016.12.008

[kiaa062-B32] González M , Delgado-Requerey V, Ferrández J, Serna A, Cejudo FJ (2019) Insights into the function of NADPH thioredoxin reductase C (NTRC) based on identification of NTRC-interacting proteins in vivo. J Exp Bot 70**:** 5787–5798 31294455 10.1093/jxb/erz326 PMC6812714

[kiaa062-B33] Hashida SN , Miyagi A, Nishiyama M, Yoshida K, Hisabori T, Kawai-Yamada M (2018) Ferredoxin/thioredoxin system plays an important role in the chloroplastic NADP status of Arabidopsis. Plant J 95**:** 947–960 29920827 10.1111/tpj.14000

[kiaa062-B34] Hernández-Verdeja T , Vuorijoki L, Strand A (2020) Emerging from the darkness: interplay between light and plastid signaling during chloroplast biogenesis. Physiol Plant 176**:** 967–976 10.1111/ppl.13100 32222991

[kiaa062-B35] Holmgren A (1995) Thioredoxin structure and mechanism: conformational changes on oxidation of the active-site sulfhydryls to a disulfide. Structure 3**:** 239–243 7788289 10.1016/s0969-2126(01)00153-8

[kiaa062-B36] Ishiga Y , Ishiga T, Ikeda Y, Matsuura T, Mysore KS (2016) NADPH-dependent thioredoxin reductase C plays a role in nonhost disease resistance against *Pseudomonas syringae* pathogens by regulating chloroplast-generated reactive oxygen species. PeerJ 4**:** e1938 27168965 10.7717/peerj.1938 PMC4860297

[kiaa062-B37] Ishiga Y , Ishiga T, Wangdi T, Mysore KS, Uppalapati SR (2012) NTRC and chloroplast-generated reactive oxygen species regulate *Pseudomonas syringae* pv. tomato disease development in tomato and Arabidopsis. Mol Plant Microb Interact 25**:** 294–306 10.1094/MPMI-05-11-0130 22112219

[kiaa062-B38] Jacquot JP , Eklund H, Rouhier N, Schürmann P (2009) Structural and evolutionary aspects of thioredoxin reductases in photosynthetic organisms. Trends Plant Sci 14**:** 336–343 19446492 10.1016/j.tplants.2009.03.005

[kiaa062-B39] Jarvis P , López-Juez E (2013) Biogenesis and homeostasis of chloroplasts and other plastids. Nat Rev Mol Cell Biol 14**:** 787–802 24263360 10.1038/nrm3702

[kiaa062-B40] Kindgren P , Kremnev D, Blanco NE, de Dios Barajas López J, Fernández AP, Tellgren-Roth C, Kleine T, Small I, Strand A (2012) The plastid redox insensitive 2 mutant of Arabidopsis is impaired in PEP activity and high light-dependent plastid redox signalling to the nucleus. Plant J 70**:** 279–291 22211401 10.1111/j.1365-313X.2011.04865.x

[kiaa062-B41] Kirchsteiger K , Pulido P, González M, Cejudo FJ (2009) NADPH thioredoxin reductase C controls the redox status of chloroplast 2-Cys peroxiredoxins in *Arabidopsis thaliana*. Mol Plant 2**:** 298–307 19825615 10.1093/mp/ssn082

[kiaa062-B42] Kirchsteiger K , Ferrández J, Pascual MB, González M, Cejudo FJ (2012) NADPH thioredoxin reductase C is localized in plastids of photosynthetic and nonphotosynthetic tissues and is involved in lateral root formation in Arabidopsis. Plant Cell 24**:** 1534–1548 22505729 10.1105/tpc.111.092304 PMC3398562

[kiaa062-B43] Khorobrykh S , Havurinne V, Mattila H, Tyystjärvi E (2020) Oxygen and ROS in photosynthesis. Plants 9**:** 91 10.3390/plants9010091 PMC7020446 31936893

[kiaa062-B44] Knuesting J , Scheibe R (2018) Small molecules govern thiol redox switches. Trends Plant Sci 23**:** 769–782 30149854 10.1016/j.tplants.2018.06.007

[kiaa062-B45] Larkin RM (2016) Tetrapyrrole signaling in plants. Front Plant Sci 7**:** 1586 27807442 10.3389/fpls.2016.01586 PMC5069423

[kiaa062-B46] Larkin RM , Alonso JM, Ecker JR, Chory J (2003) GUN4, a regulator of chlorophyll synthesis and intracellular signaling. Science 299**:** 902–906 12574634 10.1126/science.1079978

[kiaa062-B47] Leegood RC , Walker DA (1980) Regulation of fructose-1,6-bisphosphatase activity in intact chloroplasts. Studies of the mechanism of inactivation. Biochim Biophys Acta 593**:** 362–370 6263323 10.1016/0005-2728(80)90073-0

[kiaa062-B48] Lepistö A , Kangasjärvi S, Luomala EM, Brader G, Sipari N, Keränen M, Keinänen M, Rintamäki E (2009) Chloroplast NADPH–thioredoxin reductase interacts with photoperiodic development in Arabidopsis. Plant Physiol 149**:** 1261–1276 19151130 10.1104/pp.108.133777 PMC2649390

[kiaa062-B49] Lepistö A , Pakula E, Toivola J, Krieger-Liszkay A, Vignols F, Rintamäki E (2013) Deletion of chloroplast NADPH-dependent thioredoxin reductase results in inability to regulate starch synthesis and causes stunted growth under short-day photoperiods. J Exp Bot 64**:** 3843–3854 23881397 10.1093/jxb/ert216 PMC3745738

[kiaa062-B50] Liebthal M , Maynard D, Dietz KJ (2018) Peroxiredoxins and redox signaling in plants. Antioxid Redox Signal 28**:** 609–624 28594234 10.1089/ars.2017.7164 PMC5806080

[kiaa062-B51] Liebthal M , Schuetze J, Dreyer A, Mock H-P, Dietz K-J (2020) Redox conformation-specific protein–protein interactions of 2-Cys peroxiredoxins in Arabidopsis. Antioxidants 9**:** 515 10.3390/antiox9060515 PMC7346168 32545358

[kiaa062-B52] Lim S-L , Voon CP, Guan X, Yang Y, Gardeström P, Lim BL (2020) In planta study of photosynthesis and photorespiration using NADPH and NADH/NAD^+^ fluorescence sensor protens. Nat Commun 11**:** 3238 32591540 10.1038/s41467-020-17056-0 PMC7320160

[kiaa062-B53] Maruta T , Sawa Y, Shigeoka S, Ishikawa T (2016) Diversity and evolution of ascorbate peroxidase functions in chloroplasts: more than just a classical antioxidant enzyme? Plant Cell Physiol 57**:** 1377–1386 26738546 10.1093/pcp/pcv203

[kiaa062-B54] Michalska J , Zauber H, Buchanan BB, Cejudo FJ, Geigenberger P (2009) NTRC links built-in thioredoxin to light and sucrose in regulating starch synthesis in chloroplasts and amyloplasts. Proc Natl Acad Sci U S A 106**:** 9908–9913 19470473 10.1073/pnas.0903559106 PMC2700983

[kiaa062-B55] Michelet L , Zaffagnini M, Morisse S, Sparla F, Pérez-Pérez ME, Francia F, Danon A, Marchand CH, Fermani S, Trost P, et al (2013) Redox regulation of the Calvin–Benson cycle: something old, something new. Front Plant Sci 4**:** 470 24324475 10.3389/fpls.2013.00470 PMC3838966

[kiaa062-B56] Mochizuki N , Brusslan JA, Larkin R, Nagatani A, Chory J (2001) Arabidopsis genomes uncoupled 5 (GUN5) mutant reveals the involvement of Mg-chelatase H subunit in plastid-to-nucleus signal transduction. Proc Natl Acad Sci U S A 98**:** 2053–2058 11172074 10.1073/pnas.98.4.2053 PMC29380

[kiaa062-B57] Montrichard F , Alkhalfioui F, Yano H, Vensel WH, Hurkman WJ, Buchanan BB (2009) Thioredoxin targets in plants: the first 30 years. J Proteomics 72**:** 452–474 19135183 10.1016/j.jprot.2008.12.002

[kiaa062-B58] Moon JC , Jang HH, Chae HB, Lee JR, Lee SY,, Jung YJ, Shin MR, Lim HS, Chung WS,, Yun DJ, et al (2006) The C-type Arabidopsis thioredoxin reductase ANTR-C acts as an electron donor to 2-Cys peroxiredoxins in chloroplasts. Biochem Biophys Res Commun 348**:** 478–484 16884685 10.1016/j.bbrc.2006.07.088

[kiaa062-B59] Nájera VA , González MC, Pérez-Ruiz JM,, Cejudo FJ (2017) An event of alternative splicing affects the expression of the NTRC gene, encoding NADPH-thioredoxin reductase C, in seed plants. Plant Sci 258**:** 21–28 28330560 10.1016/j.plantsci.2017.02.001

[kiaa062-B60] Naranjo B , Mignee C, Krieger-Liszkay A, Hornero-Méndez D, Gallardo-Guerrero L, Cejudo FJ, Lindahl M (2016) The chloroplast NADPH thioredoxin reductase C, NTRC, controls non-photochemical quenching of light energy and photosynthetic electron transport in Arabidopsis. Plant Cell Environ 39**:** 804–822 26476233 10.1111/pce.12652

[kiaa062-B61] Navrot N , Collin V, Gualberto J, Gelhaye E, Hirasawa M, Rey P, Knaff DB, Issakidis E, Jacquot J-P, Rouhier N (2006) Plant glutathione peroxidases are functional peroxiredoxins distributed in several subcellular compartments and regulated during biotic and abiotic stresses. Plant Physiol 142**:** 1364–1379 17071643 10.1104/pp.106.089458 PMC1676047

[kiaa062-B62] Nikkanen L , Rintamäki E (2019) Chloroplast thioredoxin systems dynamically regulate photosynthesis in plants. Biochem J 476**:** 1159–1172 30988137 10.1042/BCJ20180707 PMC6463390

[kiaa062-B63] Nikkanen L , Toivola J, Rintamäki E (2016) Crosstalk between chloroplast thioredoxin systems in regulation of photosynthesis. Plant Cell Environ 39**:** 1691–1705 26831830 10.1111/pce.12718

[kiaa062-B64] Ojeda V , Pérez-Ruiz JM, González MC, Nájera VA, Sahrawy M, Serrato AJ, Geigenberger P, Cejudo FJ (2017) NADPH thioredoxin reductase C and thioredoxins act concertedly in seedling development. Plant Physiol 174**:** 1436–1448 28500266 10.1104/pp.17.00481 PMC5490916

[kiaa062-B65] Ojeda V , Pérez-Ruiz JM, Cejudo FJ (2018a) The NADPH-dependent thioredoxin reductase C-2-Cys peroxiredoxin redox system modulates the activity of thioredoxin *x* in Arabidopsis chloroplasts. Plant Cell Physiol 59**:** 2155–2164 30011001 10.1093/pcp/pcy134

[kiaa062-B66] Ojeda V , Pérez-Ruiz JM, Cejudo FJ (2018b) 2-Cys peroxiredoxins participate in the oxidation of chloroplast enzymes in the dark. Mol Plant 11**:** 1377–1388 30292682 10.1016/j.molp.2018.09.005

[kiaa062-B67] Pascual MB , Mata-Cabana A, Florencio FJ, Lindahl M, Cejudo FJ (2010) Overoxidation of 2-Cys peroxiredoxin in prokaryotes: cyanobacterial 2-Cys peroxiredoxins sensitive to oxidative stress. J Biol Chem 285**:** 34485–34492 20736168 10.1074/jbc.M110.160465 PMC2966063

[kiaa062-B68] Passaia G , Queval G, Bai J, Margis-Pinheiro M, Foyer CH (2014) The effects of redox controls mediated by glutathione peroxidases on root architecture in *Arabidopsis thaliana*. J Exp Bot 65**:** 1403–1413 24470466 10.1093/jxb/ert486 PMC3969529

[kiaa062-B69] Peltier J-B , Cai Y, Sun Q, Zabrouskov V, Giacomelli L, Rudella A, Ytterberg AJ, Rutschow H, van Wijk KJ (2006) The oligomeric stromal proteome of *Arabidopsis thaliana* chloroplasts. Mol Cell Proteomics 5**:** 114–133 16207701 10.1074/mcp.M500180-MCP200

[kiaa062-B70] Pérez-Ruiz JM , Spínola MC, Kirchsteiger K, Moreno J, Sahrawy M,, Cejudo FJ (2006) Rice NTRC is a high-efficiency redox system for chloroplast protection against oxidative damage. Plant Cell 18**:** 2356–2368 16891402 10.1105/tpc.106.041541 PMC1560923

[kiaa062-B71] Pérez-Ruiz JM , Cejudo FJ (2009) A proposed reaction mechanism for rice NADPH thioredoxin reductase C, an enzyme with protein disulfide reductase activity. FEBS Lett 583**:** 1399–1402 19345687 10.1016/j.febslet.2009.03.067

[kiaa062-B72] Pérez-Ruiz JM , Guinea M, Puerto-Galán L, Cejudo FJ (2014) NADPH thioredoxin reductase C is involved in redox regulation of the Mg-chelatase I subunit in *Arabidopsis thaliana* chloroplasts. Mol Plant 7**:** 1252–1255 24658415 10.1093/mp/ssu032

[kiaa062-B73] Pérez-Ruiz JM , Naranjo B, Ojeda V, Guinea M, Cejudo FJ (2017) NTRC-dependent redox balance of 2-Cys peroxiredoxins is needed for optimal function of the photosynthetic apparatus. Proc Natl Acad Sci U S A 114**:** 12069–12074 29078290 10.1073/pnas.1706003114 PMC5692536

[kiaa062-B74] Perkins A , Nelson KJ, Parsonage D, Poole LB, Karplus PA (2015) Peroxiredoxins: guardians against oxidative stress and modulators of peroxide signaling. Trends Biochem Sci 40**:** 435–445 26067716 10.1016/j.tibs.2015.05.001 PMC4509974

[kiaa062-B75] Peter E , Grimm B (2009) GUN4 is required for posttranslational control of plant tetrapyrrole biosynthesis. Mol Plant 2**:** 1198–1210 19995725 10.1093/mp/ssp072

[kiaa062-B76] Pfannschmidt T , Blanvillain R, Merendino L, Courtois F, Chevalier F, Liebers M, Grubler B, Hommel E, Lerbs-Mache S (2015) Plastid RNA polymerases: orchestration of enzymes with different evolutionary origins controls chloroplast biogenesis during the plant life cycle. J Exp Bot 66**:** 6957–6973 26355147 10.1093/jxb/erv415

[kiaa062-B77] Pilon M , Ravet K, Tapken W (2011) The biogenesis and physiological function of chloroplast superoxide dismutases. Biochim Biophys Acta 1807**:** 989–998 21078292 10.1016/j.bbabio.2010.11.002

[kiaa062-B78] Pinnola A , Bassi R (2018) Molecular mechanisms involved in plant photoprotection. Biochem Soc Trans 46**:** 467–482 29666217 10.1042/BST20170307

[kiaa062-B79] Puerto-Galán L , Pérez-Ruiz JM, Ferrández J, Cano B, Naranjo B, Nájera VA, González M, Lindahl AM, Cejudo FJ (2013) Overoxidation of chloroplast 2-Cys peroxiredoxins: balancing toxic and signaling activities of hydrogen peroxide. Front Plant Sci 4**:** 310 23967002 10.3389/fpls.2013.00310 PMC3746178

[kiaa062-B80] Puerto-Galán L , Pérez-Ruiz JM, Guinea M, Cejudo FJ (2015) The contribution of NADPH thioredoxin reductase C (NTRC) and sulfiredoxin to 2-Cys peroxiredoxin overoxidation in *Arabidopsis thaliana* chloroplasts. J Exp Bot 66**:** 2957–2966 25560178 10.1093/jxb/eru512 PMC4423512

[kiaa062-B81] Pulido P , Spínola MC, Kirchsteiger K, Guinea M, Pascual MB, Sahrawy M, Sandalio LM, Dietz KJ, González M,, Cejudo FJ (2010) Functional analysis of the pathways for 2-Cys peroxiredoxin reduction in *Arabidopsis thaliana* chloroplasts. J Exp Bot 61**:** 4043–4054 20616155 10.1093/jxb/erq218 PMC2935875

[kiaa062-B82] Richter AS , Peter E, Rothbart M, Schlicke H, Toivola J, Rintamäki E, Grimm B (2013) Posttranslational influence of NADPH-dependent thioredoxin reductase C on enzymes in tetrapyrrole synthesis. Plant Physiol 162**:** 63–73 23569108 10.1104/pp.113.217141 PMC3641230

[kiaa062-B83] Richter AS , Pérez-Ruiz JM, Cejudo FJ, Grimm B (2018) Redox control of chlorophyll biosynthesis mainly depends on thioredoxins. FEBS Lett 592**:** 3111–3115 30076598 10.1002/1873-3468.13216

[kiaa062-B84] Rouhier N , Cerveau D, Couturier J, Reichheld JP, Rey P (2015) Involvement of thiol-based mechanisms in plant development. Biochim Biophys Acta 1850**:** 1479–1496 25676896 10.1016/j.bbagen.2015.01.023

[kiaa062-B85] Schürmann P , Buchanan BB (2008) The ferredoxin/thioredoxin system of oxygenic photosynthesis. Antioxid Redox Signal 10**:** 1235–1274 18377232 10.1089/ars.2007.1931

[kiaa062-B86] Selinski J , Scheibe R (2019) Malate valves: old shuttles with new perspectives. Plant Biol (Stuttg) 21 (Suppl 1)**:** 21–30 29933514 10.1111/plb.12869 PMC6586076

[kiaa062-B87] Serrato AJ , Pérez-Ruiz JM, Spínola MC, Cejudo FJ (2004) A novel NADPH thioredoxin reductase, localized in the chloroplast, which deficiency causes hypersensitivity to abiotic stress in *Arabidopsis thaliana*. J Biol Chem 279**:** 43821–43827 15292215 10.1074/jbc.M404696200

[kiaa062-B88] Spínola MC , Pérez-Ruiz JM, Pulido P, Kirchsteiger K, Guinea M, González M,, Cejudo FJ (2008) NTRC new ways of using NADPH in the chloroplast. Physiol Plant 133**:** 516–524 18346073 10.1111/j.1399-3054.2008.01088.x

[kiaa062-B89] Stenbaek A , Hansson A, Wulff RP, Hansson M, Dietz KJ, Jensen PE (2008) NADPH-dependent thioredoxin reductase and 2-Cys peroxiredoxins are needed for the protection of Mg-protoporphyrin monomethyl ester cyclase. FEBS Lett 582**:** 2773–2778 18625226 10.1016/j.febslet.2008.07.006

[kiaa062-B90] Stocker S , Maurer M, Ruppert T, Dick TP (2017) A role for 2-Cys peroxiredoxins in facilitating cytosolic protein thiol oxidation. Nat Chem Biol 14**:** 148–155 29251718 10.1038/nchembio.2536 PMC5863949

[kiaa062-B91] Susek RE , Ausubel FM, Chory J (1993) Signal transduction mutants of Arabidopsis uncouple nuclear CAB and RBCS gene expression from chloroplast development. Cell 74**:** 787–799 7690685 10.1016/0092-8674(93)90459-4

[kiaa062-B92] Tadini L , Pesaresi P, Kleine T, Rossi F, Guljamow A, Sommer F, Mühlhaus T, Schroda M, Masiero S, Pribil M, et al (2016) GUN1 controls accumulation of the plastid ribosomal protein S1 at the protein level and interacts with proteins involved in plastid protein homeostasis. Plant Physiol 170**:** 1817–1830 26823545 10.1104/pp.15.02033 PMC4775149

[kiaa062-B93] Tanaka R , Kobayashi K, Masuda T (2011) Tetrapyrrole metabolism in *Arabidopsis thaliana*. Arabidopsis Book 9**:** e0145 22303270 10.1199/tab.0145 PMC3268503

[kiaa062-B94] Thormählen I , Meitzel T, Groysman J, Ochsner AB, von Roepenack Lahaye E, Naranjo B, Cejudo FJ, Geigenberger P (2015) Thioredoxin *f*1 and NADPH-dependent thioredoxin reductase C have overlapping functions in regulating photosynthetic metabolism and plant growth in response to varying light conditions. Plant Physiol 169**:** 1766–1786 26338951 10.1104/pp.15.01122 PMC4634086

[kiaa062-B95] Toivola J , Nikkanen L, Dahlstrom KM, Salminen TA, Lepisto A, Vignols HF, Rintamäki E (2013) Overexpression of chloroplast NADPH-dependent thioredoxin reductase in Arabidopsis enhances leaf growth and elucidates in vivo function of reductase and thioredoxin domains. Front Plant Sci 4**:** 389 24115951 10.3389/fpls.2013.00389 PMC3792407

[kiaa062-B96] Vaseghi MJ , Chibani K, Telman W, Liebthal MF, Gerken M, Schnitzer H, Mueller SM, Dietz KJ (2018) The chloroplast 2-cysteine peroxiredoxin functions as thioredoxin oxidase in redox regulation of chloroplast metabolism. Elife 7**:** e38194 30311601 10.7554/eLife.38194 PMC6221545

[kiaa062-B97] Vogel MO , Moore M, Konig K, Pecher P, Alsharafa K, Lee J, Dietz KJ (2014) Fast retrograde signaling in response to high light involves metabolite export, MITOGEN-ACTIVATED PROTEIN KINASE6, and AP2/ERF transcription factors in Arabidopsis. Plant Cell 26**:** 1151–1165 24668746 10.1105/tpc.113.121061 PMC4001375

[kiaa062-B98] Wang P , Liu J, Liu B, Da Q, Feng D, Su J, Zhang Y, Wang J, Wang HB (2014) Ferredoxin:thioredoxin reductase is required for proper chloroplast development and is involved in the regulation of plastid gene expression in *Arabidopsis thaliana*. Mol Plant 7**:** 1586–1590 24890758 10.1093/mp/ssu069

[kiaa062-B99] Waszczak C , Akter S, Jacques S, Huang J, Messens J, Van Breusegem F (2015) Oxidative post-translational modifications of cysteine residues in plant signal transduction. J Exp Bot 66**:** 2923–2934 25750423 10.1093/jxb/erv084

[kiaa062-B100] Waszczak C , Carmody M, Kangasjarvi J (2018) Reactive oxygen species in plant signaling. Annu Rev Plant Biol 69**:** 209–236 29489394 10.1146/annurev-arplant-042817-040322

[kiaa062-B101] Wimmelbacher M , Börnke F (2014) Redox activity of thioredoxin *z* and fructokinase-like protein 1 is dispensable for autotrophic growth of *Arabidopsis thaliana*. J Exp Bot 65**:** 2405–2413 24659486 10.1093/jxb/eru122 PMC4036507

[kiaa062-B102] Wood ZA , Poole LB, Karplus PA (2003) Peroxiredoxin evolution and the regulation of hydrogen peroxide signaling. Science 300**:** 650–653 12714747 10.1126/science.1080405

[kiaa062-B103] Wu GZ , Meyer EH, Richter AS, Schuster M, Ling Q, Schottler MA, Walther D, Zoschke R, Grimm B, Jarvis RP, et al (2019) Control of retrograde signalling by protein import and cytosolic folding stress. Nat Plants 5**:** 525–538 31061535 10.1038/s41477-019-0415-y

[kiaa062-B104] Yoshida K , Hara A, Sugiura K, Fukaya Y, Hisabori T (2018) Thioredoxin-like2/2-Cys peroxiredoxin redox cascade supports oxidative thiol modulation in chloroplasts. Proc Natl Acad Sci U S A 115**:** E8296–E8304 30104347 10.1073/pnas.1808284115 PMC6126724

[kiaa062-B105] Yoshida K , Hisabori T (2016) Two distinct redox cascades cooperatively regulate chloroplast functions and sustain plant viability. Proc Natl Acad Sci U S A 115**:** E8296–E8304 10.1073/pnas.1604101113 PMC4941451 27335455

[kiaa062-B106] Young D , Pedre B, Ezerina D, De Smet B, Lewandowska A, Tossounian MA, Bodra N, Huang J, Astolfi Rosado L, Van Breusegem F, et al (2019) Protein promiscuity in H_2_O_2_ signaling. Antioxid Redox Signal 30**:** 1285–1324 29635930 10.1089/ars.2017.7013

[kiaa062-B108] Zaffagnini M , Fermani S, Marchand CH, Costa A, Sparla F, Rouhier N, Geigenberger P, Lemaire SD, Trost P (2019) Redox homeostasis in photosynthetic organisms: novel and established thiol-based molecular mechanisms. Antioxid Redox Signal 31**:** 155–210 30499304 10.1089/ars.2018.7617

[kiaa062-B110] Zandalinas SI , Fichman Y, Devireddy AR, Sengupta S, Azad RK, Mittler R (2020) Systemic signaling during abiotic stress combination in plants. Proc Natl Acad Sci U S A 117**:** 13810–13820 32471943 10.1073/pnas.2005077117 PMC7306788

